# Enhancing Knee Joint Proprioception in Healthy Adults Through Exergame Training With Augmented Feedback: Randomized Controlled Pilot Trial

**DOI:** 10.2196/78525

**Published:** 2026-02-26

**Authors:** Yiling Zhang, Luis Felipe García Arias, Hans Timmerman, Ming Cao, Elisabeth Wilhelm

**Affiliations:** 1 Engineering and Technology Institute Faculty of Science and Engineering Universtiy of Groningen Groningen The Netherlands; 2 Department of Anesthesiology Pain Center University Medical Center Groningen Groningen The Netherlands

**Keywords:** home-based exergame, augmented feedback, auditory feedback, knee proprioception, Inertial Measurement Unit

## Abstract

**Background:**

Proprioception training is essential for restoring knee function in several medical conditions. Open kinetic chain (OKC) and closed kinetic chain (CKC) exercises are used in active movement interventions to enhance proprioception. Exergames, supported by wearable sensors, offer a solution by providing real-time feedback. Auditory feedback (AF) embedded in the serious game training has shown benefits in upper limb rehabilitation compared to visual feedback (VF) alone. However, the potential of AF in exergames that provide knee training is not known.

**Objective:**

This study presents an exergame platform aimed at enhancing knee joint proprioception through stretching and squatting exercises. The platform allows to provide feedback in 2 modes, namely, VF only and a combination of AF and VF. The AF indicates the joint position by adjusting the loudness of the sound. The VF maps the motion of the lower limb into the game space, where this information is used to control a game object. A randomized controlled trial with 14 participants compared AF to VF only. The hypothesis was that AF would improve knee joint position accuracy, enhancing neuromuscular coordination and lower limb stability.

**Methods:**

A randomized controlled trial was conducted within 14 healthy volunteers to test the exergames for knee joint motor learning using augmented feedback. All participants were required to do a pretest consisting of half and full squats and two tasks, in which participants were asked to reproduce a 45-degree knee bend and to stretch their knee fully. After that, participants played 4 rounds of each of the 2 exergames. Then the tasks of the pretest were repeated. A 1-sided Mann-Whitney *U* test was conducted for answering whether AF has a positive effect on the ability of participants to accurately control the knee joint angle. In addition, we calculated the muscle synergies participants used to complete the exercises. Subjective gaming experience was assessed using the Intrinsic Motivation Inventory and the User Experience Questionnaire.

**Results:**

A total of 14 participants were recruited, including 7 in the experimental group with AF, and 7 in the control group only with VF. The result of the Mann-Whitney *U* test demonstrated that augmented feedback improved knee joint accuracy compared to VF in both the CKC (statistics=41.0; *P*=.04) and OKC (statistics=42.0; *P*=.03) tasks. Additionally, muscle synergy analysis revealed high consistency in different muscle synergy patterns between groups across both game types.

**Conclusions:**

Augmented feedback significantly enhanced knee joint motor learning performance (reflected in the knee joint angle positioning ability) in both CKC and OKC exergame training. Consistent muscle synergy patterns across participants show that the developed exergames are suitable for knee training. Studies in patient populations are needed to establish whether the games could be used in lower limb rehabilitation.

**Trial Registration:**

ClinicalTrials.gov NCT07141290; https://clinicaltrials.gov/study/NCT07141290

## Introduction

### Background and Rationale

Proprioceptive training has been reported to be beneficial in restoring knee joint function among clinical populations and athletes. For example, rehabilitation by means of physical therapy can restore this proprioception in patients with knee osteoarthritis [1].

According to Aman et al [2], proprioceptive training can be further divided into 5 types. These are active movement and balance training, passive movement training, somatosensory stimulation training, somatosensory discrimination training, and combined and multiple system training. The meta-analysis does not come to a clear conclusion on which of these training types is superior. It also highlights that one major limitation of the field is that there is no clear definition of the term proprioceptive training. To overcome this, the authors suggest that this term should be reserved for studies that train only based on somatosensory signals while excluding other modalities such as vision [2]. Winter et al [3] suggest a broader definition that includes any intervention aiming to improve proprioception with the ultimate goal to enhance motor function. This definition is the one that applies to most serious games, as they usually involve visual feedback (VF). Winter et al [3] also concluded that active movement interventions are an important aspect of proprioceptive training. Therefore, serious games that contain active movement interventions could be beneficial to improve proprioception.

In physiotherapy, active movements such as open kinetic chain (OKC) and closed kinetic chain (CKC) exercises are used to strengthen the muscles of the knee joint [4]. OKC exercises consist of free movement of the distal joint in space without any loading, such as seated leg extensions, terminal knee extension exercises, hamstring curls, and calf pumps [5]. OKC exercises isolate specific muscle groups, enabling precise, targeted strengthening. Furthermore, OKC exercises can increase the range of motion in patients with anterior cruciate ligament injury [6]. In contrast to OKC exercises, CKC exercises fix or load a distal segment, thereby prohibiting free movement of that segment [5]. Prominent examples of CKC exercises are squats and lunges. CKC exercises promote the cocontraction of muscles to stabilize and control joint movements [6].

In the rehabilitation context, patients ideally would train 3 to 7 days per week. However, due to the lack of physiotherapists, several sessions need to be carried out unsupervised. Exergames are an option for providing feedback, while patients with impairments of the upper and lower limbs, gait disorders, balance disorders, visual-spatial disorder, or cognitive disorder train independently [7]. Especially, video exergames become a promising option for engaging patients in rehabilitative exercises. As reviewed by Gelineau et al [8], for the physical rehabilitation exergames, nonspecific video game systems (eg, Nintendo Wi and Xbox Kinect) need to be combined with specific rehabilitation systems (eg, Rehabilitation Gaming System and virtual gloves) or specific rehabilitation devices (eg, Hand Mentor Pro and Polhemus 3) to achieve satisfying training results in patients with stroke [8].

The rehabilitation systems that can be combined with exergames usually contain sensors to track the participant’s motion. Inertial Measurement Units (IMUs), which measure linear acceleration and angular acceleration, can be used to monitor kinetic motion parameters [9]. In addition, some devices use electromyography (EMG) to extract information on the activation of different muscle groups [10,11]. Metrics derived from these different sensor modalities can be used to calculate, analyze, and predict whether a user accomplishes the prescribed exercises. The use of sensorized systems makes it possible to inform exergame users about the knowledge of results (KOR), which can be used as extrinsic feedback. The KOR is defined as information about the measured error between the terminal limb position and the given target position [12]. Traditionally, most video games use a visual representation to inform users about the KOR.

Augmented feedback has been suggested as a strategy to enhance the performance of rehabilitation training with exergames. Among the different feedback modalities that have been suggested for motor learning, auditory feedback (AF), which converts movement-related data into sound, has been successfully used in tasks with low functional task complexity. Sounds can either be used to warn participants (alarms), to make them more aware of their movements (motion sonification), or to amplify errors (error amplification) [13]. The decrease in performance when feedback is removed is smaller in AF as compared to VF, indicating that participants who receive AF do not become dependent on the presence of the feedback [14].

For proprioceptive training in older adults, a combination of a commercially available stepping game (Stomp it) and a game in which participants have to kick a virtual ball (Sport Kinect target kick) or prevent a ball from entering the goal (Sport Kinect goal keeper game) has been investigated. These games mainly focus on OKC tasks. Furthermore, the games that are related to soccer playing require a mix of knee movement and balancing. The feedback in the games is mostly VF. To assess the effect of an 8-week training program with these games, participants were asked to reproduce knee angles when seated. The instructions for the knee angle reproduction assessment were given with a tactile interface. Participants were blind folded during this procedure. Participants of the intervention group reduced the knee proprioception error from 5.2 to 3.5 for a target angle of 30. Similar significant improvements were also reported for other target angles [15]. For patients with knee osteoarthritis, a game that provides VF on CKC exercises was proposed. The exercises in this game were squatting and leg presses against an elastic Theraband. The proprioception was assessed by asking participants to reproduce a knee angle that has previously visually been demonstrated to them. For a target position of 30, the knee proprioception error improved from 6.25 to 3.5 in a training period of 6 weeks [16]. Another study in patients with osteoarthritis focused on balance games with the Nintendo Wii Fit. The games are, in general, challenging the balance of the user. As the solution the player attempts is not limited, they can contain a mix of open and closed kinematic chain motions. In accordance with its main goal, this study focused on balance-related outcome measures [17]. No study so far has systematically investigated whether the efficacy of AF is related to the type of exercise (OCK vs CKC) in lower limb training.

### Objectives

In this study, we developed a video exergame system for enhancing position accuracy through targeted training with incorporated motion tracking and augmented feedback. The system contains 2 different games, one for CKC and one for OKC exercises. The CKC game is called Bunny Game and encourages users to do half squats and deep squats. The OKC game, Fish Game, asks users to perform 4 different knee joint stretching tasks. To provide feedback, the knee joint angle was measured by IMU sensors. This information was used to adjust the position of a game object on the screen (VF) and provide motion sonification (AF).

To verify whether augmented feedback training yields better results than VF, a pilot study was conducted with 14 healthy participants, 7 in the augmented feedback group (experimental group [EG]) and 7 in the VF group (control group [CG]). All participants played both games, one with an OKC task and one with a CKC task in a randomized order. We hypothesized that participants in the EG would significantly improve knee joint position accuracy compared to the CG. In total, 7 EMG signals from the nondominant leg of the participants were collected to analyze the muscle synergies used during training.

## Methods

This paper adheres to the CONSORT (Consolidated Standards of Reporting Trials). The CONSORT checklist for this paper can be found in Multimedia Appendix 1.

### Game Development

#### CKC Exergame

**Figure 1:**
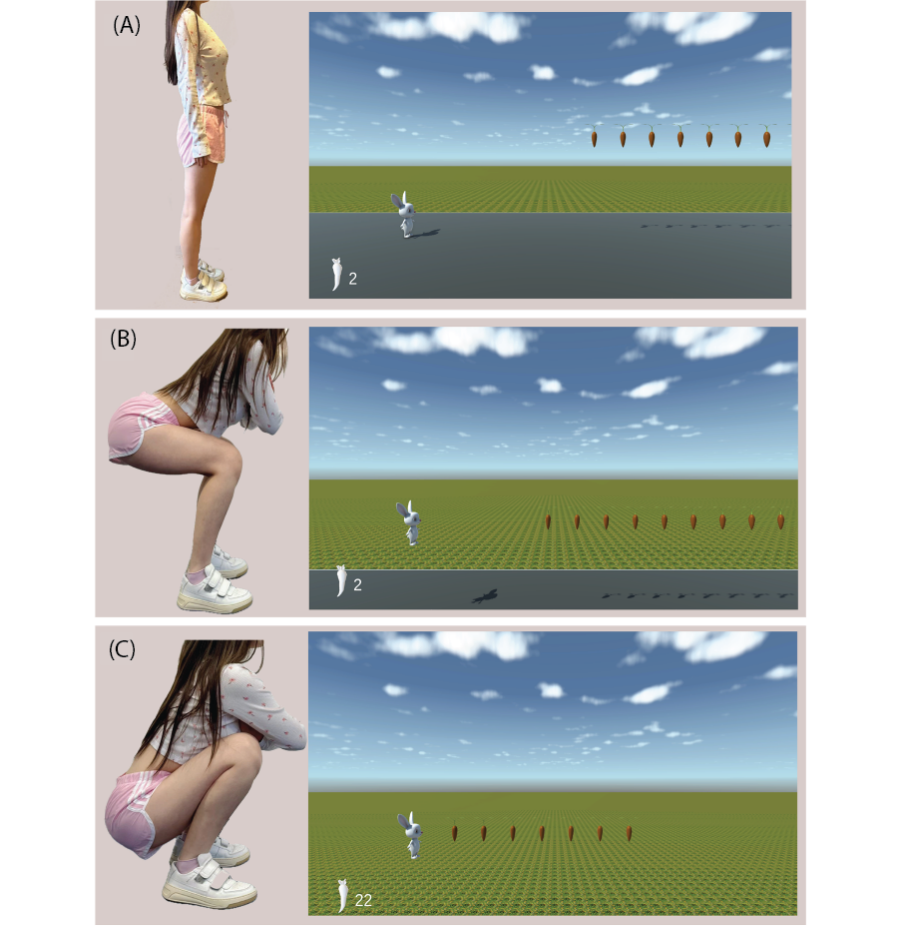
Illustration of the game with closed kinetic chain movements. (A) Preparation in stance gesture. (B) Half squat demonstration and the carrots’ height in the user interface. (C) Deep squat demonstration and the height of the carrots that should trigger this motion.

The user interface (UI) of the CKC exergame, called “Bunny Game,” is shown in Figure 1. In this game, participants performed 2 different types of squats to control the vertical position of the bunny on the screen: the deeper the squat, the higher the bunny jumps. The sonification of the position of the bunny is based on a sequence of brief, percussive tones with varying pitches. The pitch increases as the bunny’s vertical position rises. Carrots were set at 2 heights: one reachable by a half squat, as shown in Figure 1B, and the other by a deep squat, as shown in Figure 1C. When the bunny reaches a carrot, it is collected, and the score increases by 1. Each group consists of 9 carrots, requiring the player to hold the squat position for 2.5 seconds to collect all of them. Each group of carrots is followed by a 20-second gap to allow the player to stand up and rest. One round of the game contains 12 groups of carrots, thereby prompting the player to perform 12 squats per round.

#### OKC Exergame

**Figure 2:**
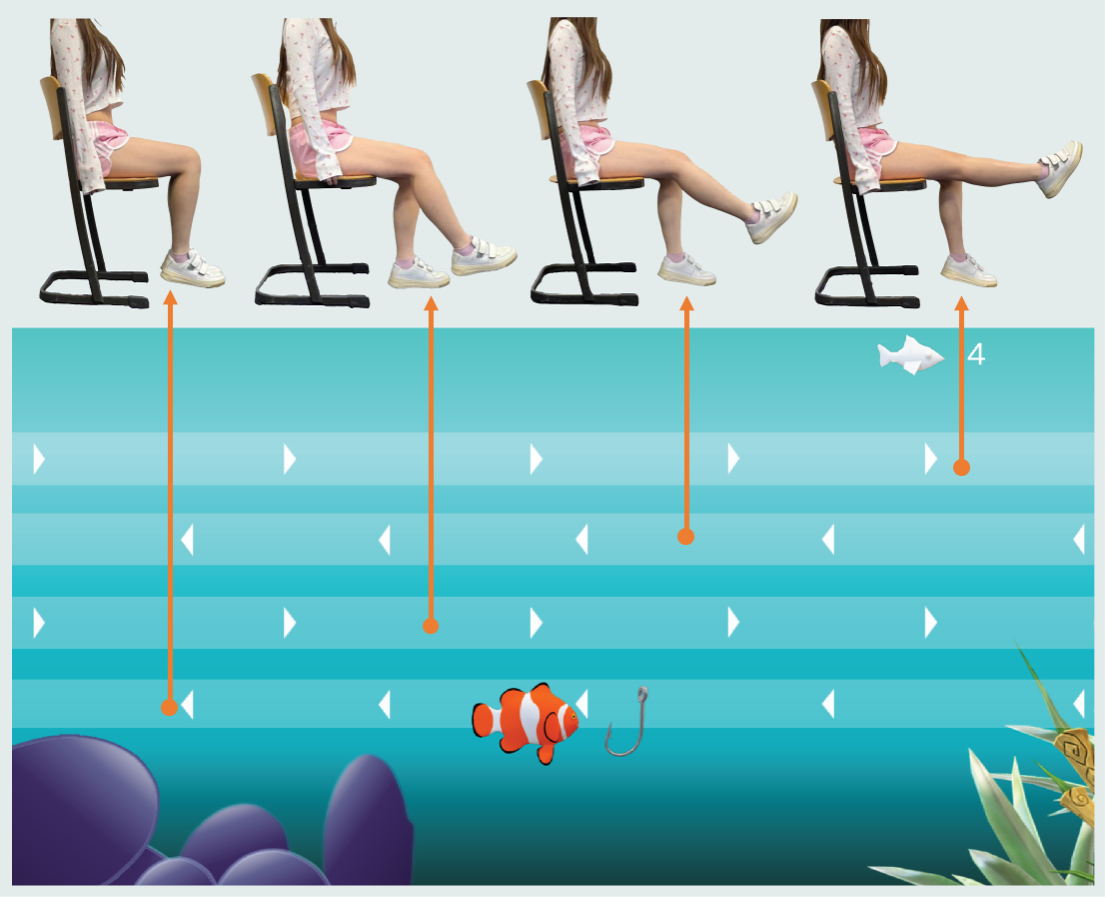
Illustration of the game with open kinetic chain motion. From left to right are the demonstrations of knee joint angles in 90°, 120°, 150°, and 180°, which correspond to 4 trials.

The UI of the stretch exergame “Fish Game” is shown in Figure 2. In this game, 12 fish appear randomly along 4 different trails, each corresponding to a specific knee stretch movement. The lowest trail matched the movement of the 90° knee joint, the second lowest trail matched the 120° knee joint bending, the second highest trail matched the 150° knee joint bending, and the highest trail matched the total stretching. The participant rotates the knee joint of the tested leg to move the hook from one trail to the other in a vertical direction. The pitch of the sonification prompt increases as the vertical position of the hook rises. If the hook stays for more than 2 seconds on the trail where a fish appears, the fish is caught, and the score increases by 1. If the fish is not caught within 10 seconds, it reaches the other side of the screen, where it disappears. The word “Miss” is shown on the screen to communicate to the player that the fish escaped.

### Auditory Feedback

The sound used in AF mode consisted of a short, percussive tone extracted from a rain sound recording, which gave a crisp, bell-like quality with natural texture. The pitch of the sonification was modulated according to the vertical position of the task object, such that the higher the bunny or hook, the louder the sound. The audio was delivered through the Unity engine, with amplitude scaled linearly from silence to the maximum nonclipping volume. The duration of 1 auditory stimulus was 0.1-0.2 seconds. After this period, the next fragment with a different pitch was played.

### Game Integration With Sensors

This knee joint training game system was developed using Unity (version 2021.3.0.f1; Unity Technologies). Both kinematic data and EMG data were collected with 9 Avanti sensors (Delsys Europe Ltd). Kinematic data were obtained from 3 Avanti sensors placed on midshank and biceps femoris (BF) muscle on the participant’s nondominant leg and the one on the collarbone region of the trunk. Muscular activity of tibialis anterior, gastrocnemius medialis, gluteus medius, gastrocnemius lateralis, peroneus longus, BF, and rectus femoris in the nondominant leg were measured with 6 Avanti sensors. The application programming interface of Delsys was used to stream the data from the IMU sensors in real time into the game environment.

### Sensor Calibration

**Figure 3:**
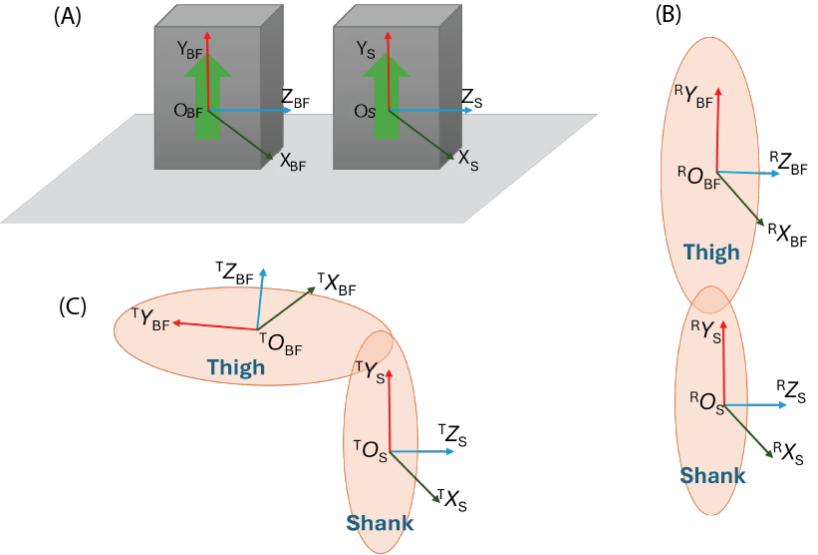
Sensor calibration process illustration: (A) sensors aligned in parallel, (B) sensors attached to the leg in an upright standing position, and (C) sensors in a movement. BF: biceps femoris.

Prior to sensor attachment, the sensors were lying next to each other in a parallel orientation on a flat desk. In this state, the sensors were connected to the game system; when the UI showed that all of the sensors were connected, the initial IMU orientation 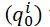 was recorded to determine the offset of individual sensors. Once all sensors started streaming data, they were attached to the legs of the participant, and the electrode location followed the SENIAM (Surface Electromyography for the Non-Invasive Assessment of Muscle) guidelines [18]. Before testing, the participants were asked to stand in an upright posture to establish a baseline orientation of the IMUs on the user’s body. This position offset was used in the kinematic analysis to prevent that difference in body shape affects the game performance. The whole calibration process is shown in Figure 3.

The data obtained during the calibration process were used for the following in-game calculations. To obtain the initial reference quaternion for knee joint angle measurements, the IMUs attached to the BF and the midshank on the nondominant leg were placed parallel to each other on a flat surface. Sensor data were then recorded for 14 seconds at a sampling frequency of 74 Hz, resulting in a buffer of 1000 unit quaternion samples, which was used to compute the reference orientation. Each quaternion in this buffer is represented as *q_i_*=(*w_i_*, *x_i_*, *y_i_*, *z_i_*). The original reference quaternion (*q*_avg_) is obtained by averaging all 1000 quaternions component-wise.

To ensure that this average remains a unit quaternion, *q_avg_* is normalized by dividing it by its magnitude:



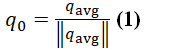



This *q*_0_ serves as the baseline orientation of the sensors.

After this calculation, all of the sensors were attached to the participant by the experimenter, with the midshank sensor placed in the middle of the shank and aligned with the nondominant leg BF sensor. The player was asked to stand upright for 14 seconds, avoiding moving. During this time, the computer calculated the offset (*q*_ref_). This information was entered in equation 2 to calculate the rotation *q*_T_ between the original quaternion *q*_original_ and the offset *q*_reference_ to account for any orientation bias due to the leg shape.



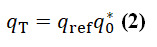



During game training, the real-time sensor orientation is computed by multiplying the measured quaternion (*q*_R_) and the offset (*q*_T_):



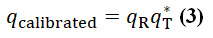



The knee angle (*q*_knee_) is defined as the angle between the calibrated coordinate system of the midshank 
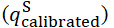
 and that of the BF of the nondominant leg 
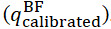
. This angle can be obtained from equation 4. The Euler angle of the knee in the Y direction is used as the game controller in games.



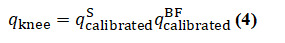



### Power Calculation

The effect size of AF was estimated to be 0.78, based on the results of Ghai et al [19] and Fujii et al [20]. The main results of this study were evaluated using a repeated-measures, between-factors ANOVA. The power was set to 80%, and an α of .05. G*Power (version 3.1.9.7; Heinrich-Heine-Universität Düsseldorf) was used to calculate the sample size a priori. This calculation indicated that a minimum number of 10 participants, including 5 participants in the EG and 5 participants in the CG, were needed to draw a conclusion. Considering a dropout rate of 30%, the recruitment target was set to 14 participants.

### Recruitment

All the participants were recruited among the employees and students of the University of Groningen. To be eligible to participate in this study, participants had to be at least 18 years of age, be healthy based on self-report, and be fluent in either English or Dutch to ensure that they understand the instructions given by the experimenter. Exclusion criteria were pregnancy, orthopedic problems, musculoskeletal disorders, prior surgery on the lower extremities, or abuse of drugs, growth hormones, anabolic steroids, or performance-enhancing substances based on self-report. Furthermore, participants with a known adhesive allergy were excluded to reduce the risk of skin irritation due to sensor attachment. To be able to attach the EMG electrodes, participants were required to wear shorts during the test. Therefore, we also had to exclude participants who were not willing to expose the skin of the legs for any reason.

### Experiment Design

#### Overview

The study design of this experiment was a randomized between-group comparison. Participants were randomly assigned to groups using a Microsoft Excel–generated sequence to minimize bias. For comparing the game effectiveness within and without AF, all participants tested both game levels in randomized order. The group the participant belongs to was predetermined via a vector generated with the random function in Excel. This vector was used to assign each participant to 1 group based on the order in which they were recruited. AF was assigned to participants in the EG, but not to the participants in the CG. To rule out confounders, the participants were assigned to the EG and CG at random, apart from the AF; both groups followed the same experimental procedure.

**Figure 4:**
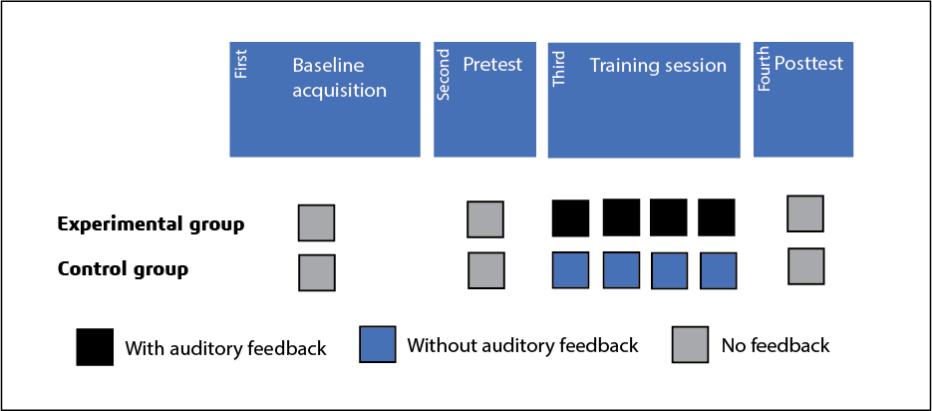
Flow chart of the study design overview and the main procedures that subjects will undergo in the course of research.

There were 4 blocks in the experiment, as shown in Figure 4.

After the sensor calibration, the pretest was performed. During the pretest, each participant performed 10 squats and 10 knee stretches. Participants during the pretest were instructed by written information on the screen on which movement they should perform. Each movement was held for approximately 5 seconds. During the squatting sequence of the pretest, the commands “half squat” or “deep squat” appeared in randomized order. During the stretching sequence of the pretest, the prompts “half stretch (45°)” or “totally stretch (0°)” appeared in random order by the script used for prompt text generation. Participants were seated for this test, and they were previously instructed to obtain a position that is in the middle between totally stretching and having their feet in a 90°. As all participants had a mathematical background, no further explanation of the instructions was necessary. Participants were not blindfolded during the test. Psychometric properties of the test are not available. Participants who were randomly allocated to start with the bunny game started the pretest with the squatting session, while participants who started with the fish game were first prompted with the stretching exercises. To initiate the pretest, the experimenter selected the “Pre-Test” scene in the game menu. This game menu consists of 3 buttons: “Bunny Game,” “Fish Game,” and “Pre-Test,” which allow players to select the different games. In the “Pre-Test” scene, the screen displays 2 buttons: “Stretch Test” and “Squat Test.” The experimenter used these buttons to select the tests in the order indicated on the random allocation list. After completing a test, the exit button returns to the main menu scene, with the Pre-Test button now labeled Post-Test. The Fish Game and Bunny Game options remain available.

After the pretest, each participant completed 4 rounds of training for both games. The training platform for the EG included AF, while for the CG, AF was turned off, leaving only VF.

In the final block, a posttest was conducted, consisting of 10 nondominant knee stretches and 10 squats. The test content and format were identical to those of the pretest. During the pre- and the posttest, participants were asked to perform 10 half squats (target knee angle [TKA]=90°) and full squats (TKA=130°). In addition, participants were asked to sit on a chair and stretch their leg 10 times with a TKA of 135° or 180°, which equals a half and full stretch, respectively.

#### Study Parameters

The main outcome was the error of position between TKA and actual knee angle (AKA) of the participant along the femoral-tibial axis. The AKA was calculated based on the IMU sensors as described in the Game Development section. The error of position was calculated by subtracting TKA from AKA (equation 5).

Error=AKA–TKA **(5)**

According to both the IMU signal and the recorded video during the test, the IMU data are segmented, with each segment representing a test movement. The error of these data segments was calculated. In each test, average error (AE) is classified by the type of movements, with 2 groups (squat test group and stretch test group) of AE in the pretest and 2 groups of AE in the posttest for each participant. The absolute mean value of each group represented the average performance of the participant in the corresponding movement during the test (average mean error [AME]), calculated by equation 6, where *n* denotes the number of times this type of movement appeared in the test, and AE denotes the average error of a movement.



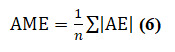



For each participant, the improvement of training was represented by the difference in performance of each group between pretest and posttest. Equation 7 expresses the calculation of the improvement for a participant in 1 movement classification, which is denoted by IE (improvement of error trajectory), AME_Pre_ denotes the absolute mean value of a group of movement errors in the pretest, and AME_Post_ denotes the absolute mean value of the group of movement error in the posttest.

IE=AME_Pre_–AME_Post_
**(7)**

#### Self-Report Instruments

Participants completed the Task Evaluation Questionnaire of Intrinsic Motivation Inventory (IMI) [21], a multidimensional measurement tool used to assess participants’ subjective motivation during game training. The following 4 subscales: interest or enjoyment, perceived competence, perceived choice, and pressure or tension, were assessed based on the Task Evaluation Questionnaire. Each subscale consisted of 5-7 items rated on a 7-point Likert scale (1=not at all true, 4=somewhat true, and 7=very true) [22].

The User Experience Questionnaire (UEQ) [23] was also filled after the experiment. Six dimensions were measured: attractiveness, perspicuity, efficiency, dependability, stimulation, and novelty. Each dimension was assessed through a set of bipolar adjective pairs (eg, “annoying-enjoyable”), rated on a 7-point scale from –3 to +3. Higher scores indicated a more positive user experience [23].

### Muscle Synergy

In this study, muscle synergy analysis was performed to examine the differences in muscle weighting patterns across different synergies during game training, following the methodology described in Hayes et al [24].

EMG signals were collected from 7 muscles (tibialis anterior, gastrocnemius medialis, gluteus medius, gastrocnemius lateralis, peroneus longus, BF, and rectus femoris) on the nondominant leg. The EMG signals were segmented based on task-specific event timestamps. For the Fish Game, segments were extracted from 2 seconds before the timestamp at which a fish was caught up to the event itself. For the Bunny Game, a valid task was defined as the collection of a complete group of 9 consecutive carrots; the corresponding segment spanned from the timestamp of the first carrot to that of the ninth in a single group. Each segment was processed with a fifth-order Butterworth low-pass filter (cutoff: 30 Hz) and high-pass filter (cutoff: 4 Hz). Following filtering, the signals were detrended by subtracting the mean and then rectified by taking the absolute value. Finally, each signal was normalized by dividing it first by its maximum value and then by the SD of the resulting signal to achieve unit variance.

Muscle weights were extracted using nonnegative matrix factorization. To determine the appropriate number of synergies required to reconstruct the original EMG signal, the variance accounted for (VAF) was calculated. For each trial, the number of synergies was identified as the minimum number for which the average VAF dropped below thresholds of 90%, 85%, and 80%, respectively, ensuring a balance between model simplicity and signal reconstruction accuracy.

### Statistics

Q-Q plots were used to test for the distribution of the data. As the data were not normally distributed, the nonparametric alternative for a repeated measures ANOVA was used. Therefore, a 1-sided Mann-Whitney *U* test was conducted for answering whether AF has a positive effect on the knee joint training based on the exergame platform. To ensure data quality and reduce the impact of extreme values, outliers were identified and removed using the *z* score method. Specifically, for each variable, *z* scores were calculated, and data points with absolute *z* scores greater than a predefined threshold (|*z*|>2.5) were regarded as extreme data and excluded from the analysis. The threshold was chosen to balance outlier detection while retaining sufficient data for robust statistical analysis.

The null hypothesis (*H*_0_) is that AF does not significantly improve trajectory error compared to without AF during stretching and squatting. The observations are squat test IE in the EG, squat test IE in the CG, stretch test IE in the EG, and stretch test IE in the CG. A significance level of α=.05 was used for hypothesis testing.

### Ethical Considerations

The study was carried out in April 2025 in the laboratories of the Discrete Technology and Production Automation group of the Engineering and Technology Institute of the University of Groningen in Groningen, The Netherlands. The study was conducted in accordance with the Declaration of Helsinki. The institutional ethics committee (Research Ethics Review Committee [CETO]) of the Faculty of Arts, University of Groningen, reviewed the experiment protocol and had no objection to the proposal (ID 93318312). Before the sessions started, participants had received an information letter describing the aim and procedures carried out during the study. They were given the opportunity to ask questions about their participation. Participants, who signed the informed consent form, were required to fill in a short demographic questionnaire and the Waterloo Footedness Questionnaire [25]. Afterward, the experimenter tested their hearing capability by Audiometer 9910 (Amplivox Ltd). Prior to data acquisition, a Data Protection Impact Assessment was carried out to ensure that the data processing is in line with European regulations. As a conclusion of this assessment, the data were stored in a secure virtual research workspace of the University of Groningen. The results are reported as median or average to ensure that individuals cannot be identified. Participants did not receive any compensation for their participation in these experiments.

## Results

### Participants

**Figure 5:**
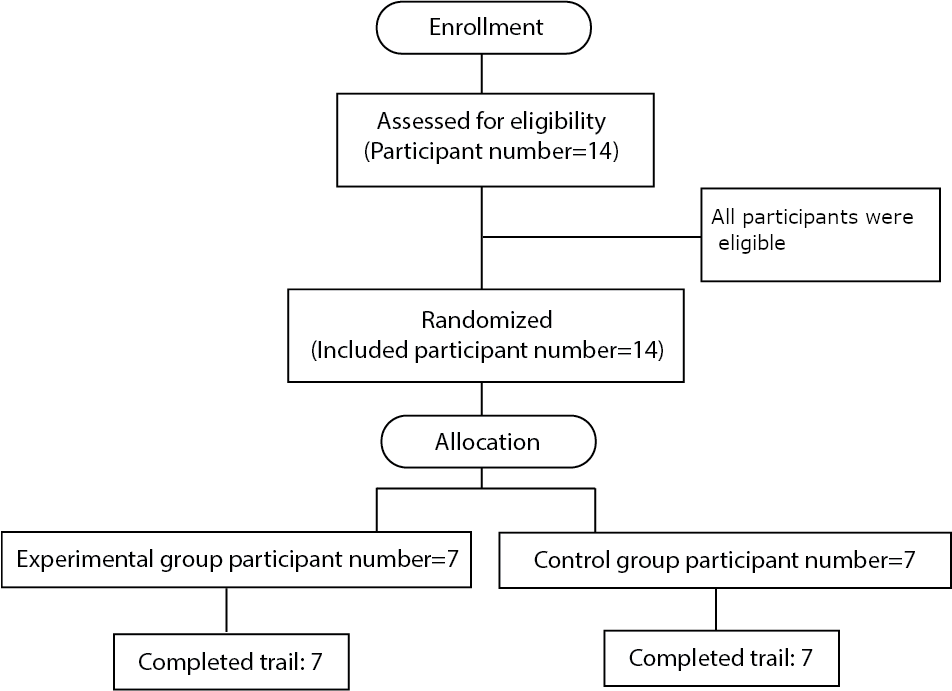
Flowchart depicting the recruitment process.

A total of 14 participants were recruited for this study: 7 participants were in the EG, and 7 participants were in the CG. All of the participants were right-leg dominant. In the augmented AF group, there were 4 female and 3 male participants; the average age was 27.58 (SD 3.09) years, and the mean BMI was 21.07 (SD 3.04) kg/m^2^. The VF group consisted of 3 female and 4 male participants; the average age was 28.69 (SD 4.17) years, and the mean BMI was 21.15 (SD 2.86) kg/m^2^. The trial ended when the recruitment target was reached. A flowchart describing the recruitment process is depicted in Figure 5.

### Kinetic Analysis

**Figure 6:**
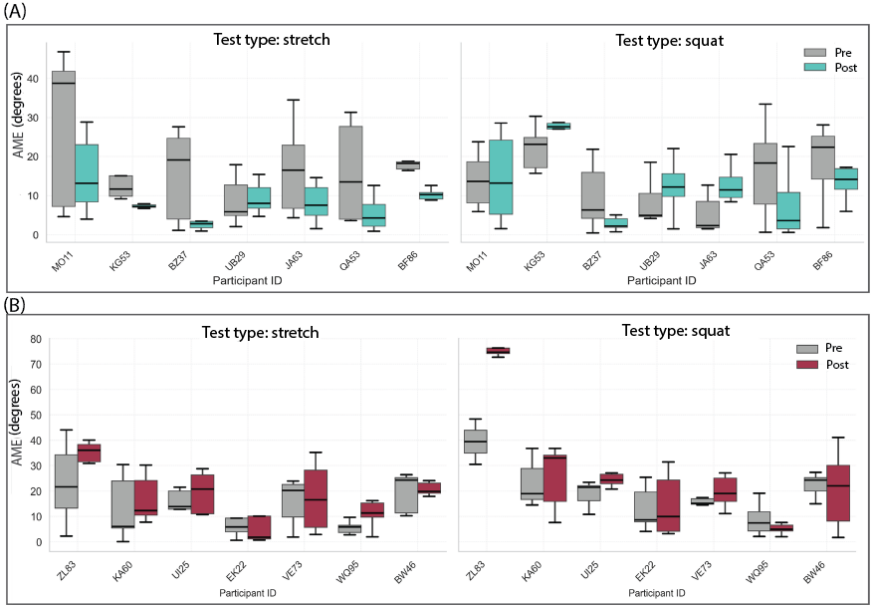
AME in the stretch and squat test in both pre- and posttest for the experimental group (EG) and the control group (CG). (A) AME of 7 participants in the EG during pre- and posttest. (B) AME of 7 participants in the CG during pre- and posttest. AME: average mean error.

Figure 6 depicts the AME during pre- and posttest per movement category and participant. Figure 6A illustrates the AME of 7 participants in the EG. In the stretch test, all EG participants showed a smaller AME, indicating an improvement in their ability to reproduce the angles. In the squat test, only 3 participants of the EG showed obvious improvement in the posttest. The other 4 participants either had a huge spread in the AME or did not improve in the posttest compared to the pretest.

Figure 6B displays the AME for 7 participants in the CG. During both the stretch and the squat test, the participants of this group showed nearly no improvement in the AME.

**Figure 7:**
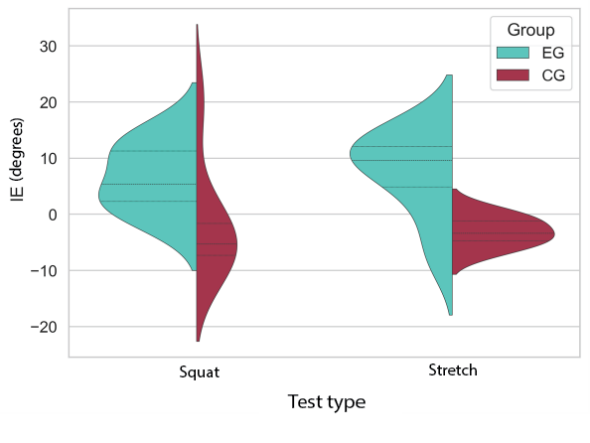
Squat and stretch test IE after intervention (with and without augmented feedback) illustration. CG: control group; EG: experimental group; IE: improvement of error trajectory.

The violin plot depicted in Figure 7 illustrates the improvement in knee joint performance for 2 test movements in both the EG and CG. Each violin plot represents the distribution of IE values within 1 category.

For the squat and stretch test groups, the mean value in the EG is higher than in the CG, indicating a notable improvement in squatting performance for the EG compared to the CG. A similar, though slightly smaller, trend is observed between the stretch test in EG and CG. The quartiles (first quartile Q1, median, and third quartile Q3) of each group of tests are depicted in Figure 7. The median improvement of the experimental group was 5.36 (IQR 2.27-11.25) and 10.46 (IQR 5.45-12.07) in the squat and stretch test, respectively. The control group achieved an IE of –5.31 (IQR –7.32 to –1.62) and –3.37 (IQR –5.73 to –1.22) in the squat and stretch test, respectively.

A 1-sided Mann-Whitney *U* test was conducted on the IE datasets, revealing a statistically significant difference between the EG and the CG (statistics=41.0; *P*=.04) for squatting and between the EG and the CG (statistics=42.0; *P*=.03) for stretching.

Participants who received augmented feedback demonstrated greater improvements in both squat and stretch tasks compared to the CG. *H*_0_ was rejected, indicating that integrating augmented AF into the exergame platform positively impacts knee joint motor training.

### Muscle Synergy

EMG data from all 14 participants (1054 exercise movements in total) were included in the muscle synergy analysis. The number of synergies required to meet the VAF thresholds varied across trials. For the 90% VAF threshold, synergy numbers ranged from 4 to 7; a total of 3 movements had 4 synergies, 98 movements had 5 synergies, 787 movements had 6 synergies, and 166 movements had 7 synergies. For 85% VAF, synergy numbers ranged from 3 to 6; a total of 3 movements had 3 synergies, 29 movements had 4 synergies, 342 movements had 5 synergies, and 682 movements had 6 synergies. For 80% VAF, synergy numbers ranged from 3 to 6; a total of 4 movements had 3 synergies, 115 movements had 4 synergies, 683 movements had 5 synergies, and 252 movements had 6 synergies. The 85% VAF threshold was selected for subsequent analysis to balance signal reconstruction quality and model simplicity, 6 synergies appeared most frequently across participants and movements, suggesting a stable underlying structure in muscle coordination.

**Figure 8:**
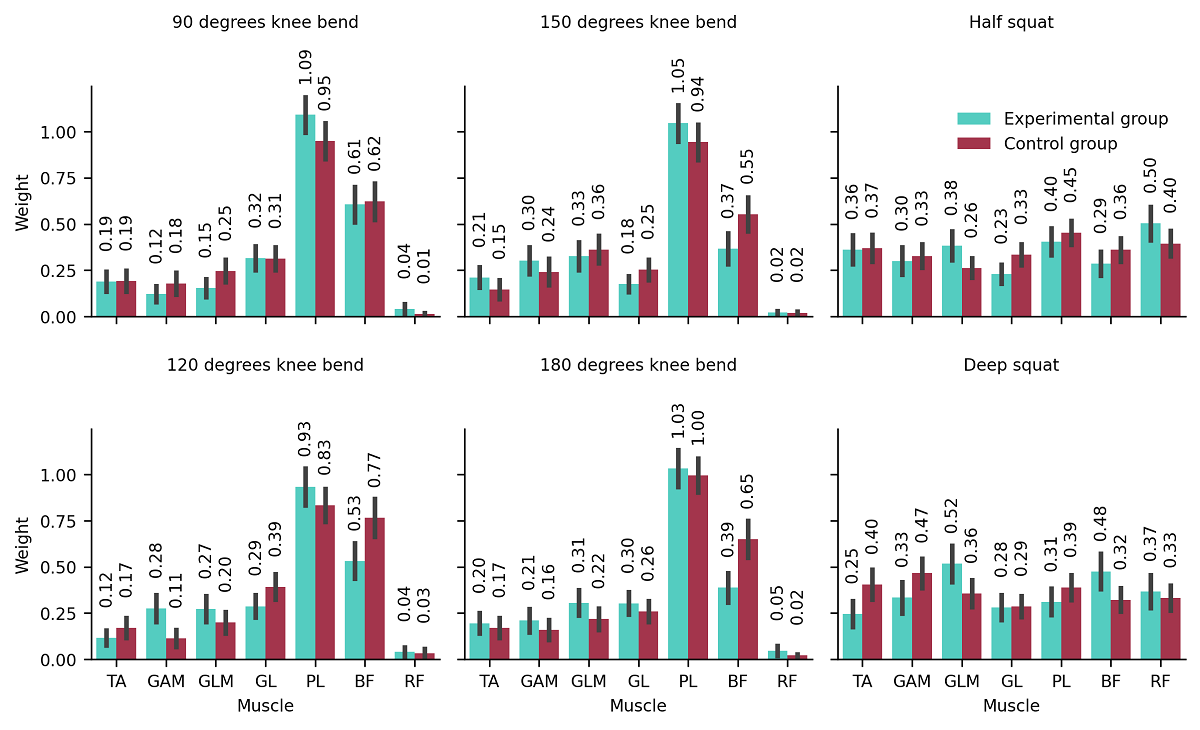
Muscle synergy analysis including tibialis anterior (TA), gastrocnemius medialis (GAM), gluteus medius (GLM), gastrocnemius lateralis (GL), peroneus longus (PL), biceps femoris (BF), and rectus femoris (RF).

One of the extracted muscle synergy weight patterns is presented in Figure 8. The complete results of the synergy analysis can be found in Multimedia Appendix 2. The first and second columns display the 4 tasks from the Fish Game, comparing 2 groups. Overall, the muscle weight distributions between the groups were largely consistent. The third column shows the 2 tasks from the Bunny Game, half squat and deep squat, with similarly consistent muscle weighting patterns observed between the 2 groups.

### Quantitative Analysis of Self-Report Measures

Table 1 summarizes the IMI subscale scores for EG and CG. The EG reported higher perceived choice, and the CG reported higher interest or enjoyment, perceived competence, and pressure or tension.

The results of UEQ were analyzed from the following 5 subscales: attractiveness, perspicuity, efficiency, dependability, stimulation, and novelty. In EG, the mean of subscales is 2.738 (SD 0.31), 2.821 (SD 0.14), 2.143 (SD 0.46), 2.500 (SD 0.27), 2.536 (SD 0.43), and 2.000 (SD 1.15), respectively. In CG, the mean (variance) of subscales is 2.357 (SD 0.70), 2.714 (SD 0.03), 2.286 (SD 0.28), 2.286 (SD 0.38), 1.929 (SD 1.31), and 1.679 (SD 2.08), respectively. According to the rating scheme of the questionnaire, these scores indicate that the experience was rated “excellent” [23]. The EG showed higher scores across all dimensions, particularly in attractiveness (mean 2.738, SD 0.31) and novelty (mean 2.000, SD 1.15), indicating a more engaging and novel user experience. Furthermore, to access the overall experience, the subscales were grouped into pragmatic and hedonic quality according to UEQ guidelines [23]. The EG scored higher on both pragmatic quality (mean 2.49) and hedonic quality (mean 2.27) compared to the CG (mean 2.43 and mean 1.80, respectively).

**Table 1:** Intrinsic Motivation Inventory subscale scores by group.

Subscale	Experimental group, mean (SD)	Control group, mean (SD)
Interest or enjoyment	35 (10)	35 (9)
Perceived competence	29 (7)	29 (7)
Perceived choice	8 (4)	8 (4)
Pressure or tension	–6 (4)	–5 (4)

## Discussion

### Main Results

The aim of this study was to explore whether augmented AF applied in motor learning during game-based knee joint exercise (involving both CKC and OKC) improves the knee angle proprioception more than VF alone. The results demonstrated that augmented AF exergame training improved the AME of the knee joint angle significantly more than VF alone. After both CKC and OKC game training, participants in the EG demonstrated significantly better knee angle positioning skills. This suggests that the benefit of auditory sonification cues can positively support both CKC and OKC knee joint tasks.

The results of self-report instruments, IMI and UEQ, showed that the experience of the participants was positive in both games. The IMI suggests that the participants felt low pressure while experiencing enjoyment, which is desired in an exergame. EG participants reported a more immersive, attractive, and novel experience compared with CG participants. These findings are consistent with prior research that supports the role of augmented feedback, especially auditory, in facilitating sensorimotor integration and enhancing motor skill learning [13]. Auditory cues provide immediate, real-time reinforcement, which can guide users’ movements, keep users engaged, and increase interactions during motor learning.

As shown in Figure 6, most participants in EG exhibited a reduction in AME during the OKC test following the augmented feedback game training. In addition, the SD of the error decreased from pretest to posttest for each participant, indicating reduced variability and more stable task performance after training. In CG, 6 of 7 participants also had a decreased SD after training. However, the mean AME did not show improvement in the OKC tasks after training. These findings are not in line with previous literature, which reports that improvements can be reached with both CKC [16] and OKC [15] exercises. However, the studies in the literature observe participants over a duration of 6 weeks, while we only investigated a single game session. Furthermore, the participants in the studies reported in the literature were older male individuals and patients with osteoarthritis. Our population was much younger and healthy. Therefore, the baseline might already have been better. Due to differences in the instructions during the assessment, a direct comparison of the absolute values is unfortunately not possible.

For the CKC test (squat test), the performance in EG after training does show smaller improvements than the OKC test result. In total, 4 of 7 participants in the EG decreased the AME after interventions, and 6 participants had a decreased SD after training. Compared to the EG, AME results of the CG show less improvement. Figure 7 demonstrates a more intuitive comparison. In EG, the improvement of knee joint positioning skills is generally greater than the CG in both squat and stretch tests. The OKC game (Fish Game) showed greater improvements in motor learning compared to the CKC game (Bunny Game).

Another observation in this study is the consistency in muscle synergy weight pattern between EG and CG across both game contexts. As shown in Figure 8, the muscle weight distributions are remarkably similar between 2 group participants, suggesting that motor control strategies in the muscle remained stable regardless of group assignment. This trend is also evident in both games. The stability in muscle synergy organization supports the use of game-based interventions as a tool for training repetitive motions.

### Limitations

Several limitations should be acknowledged in this study. First, all participants were recruited from the University of Groningen, consisting exclusively of students and researchers. This relatively homogenous sample limits the generalizability of the findings to broader populations. Second, the experiment was conducted within a single day, and the study does not capture long-term retention or progression of motor learning over time. Third, due to the nature of AF, it was not possible to blind the participants with respect to group assignment. Therefore, the subjective measures taken with the questionnaires might be influenced by the fact that participants were aware whether they were in the control or the intervention group.

Fourth, the system has not yet been tested in a clinical population. While the current results are promising, validation in clinical settings is essential to confirm the utility and effectiveness of the developed augmented AF game training system in rehabilitation contexts for the knee joint. Finally, both Fish Game and Bunny Game include 2 distinct types of motor tasks: spatial positioning of the knee joint and knee joint extreme movements. These task types were not separately analyzed in this study.

The most noticeable limitation of our paper is that participants were not blindfolded during the pre- and posttest. Therefore, the extent to which they used vision to compensate for eventual proprioceptive deficits is unknown. Due to this limitation, we can only say that the study had positive effects on motor learning in general. Future studies that test proprioception while occluding vision and apply psychometric testing methods are needed to evaluate the precise contribution of proprioception.

### Implication for Research and Future Work

Despite the limitations, this study provides a prototype of an exergame for knee joint motor learning. The result shows the benefit of augmented feedback in interactive, feedback-driven games to support effective motor learning in the lower limbs.

**Figure 9:**
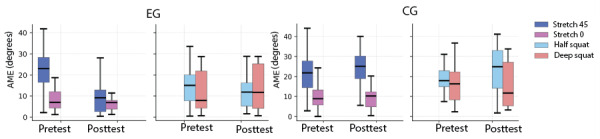
AME distribution comparison among 4 test movements. CG: control group; EG: experimental group.

Moving forward, implementing longitudinal study designs would allow for assessment of motor learning retention and the long-term impact of knee joint training based on an augmented feedback exergame. Clinical trials are also essential to explore whether the game can be used in the context of rehabilitation. Finally, further analysis should differentiate between the 2 types of motor tasks, knee joint positioning and extreme joint movement, present in the games. As shown in Figure 9, the distributions of AME between “stretch 45,” “stretch 0,” “half squat,” and “deep squat” are obviously different. For OKC tasks, knee joint positioning shows a bigger variance than the extreme joint movement. For CKC tasks, the knee joint positioning shows a smaller variance than the extreme joint movement. Investigating these categories separately could uncover task-specific motor strategies. Together, these directions can build upon the promising outcomes of this study to inform the design of adaptive, personalized game-based interventions for the knee joint motor skill learning and rehabilitation.

The tasks that we selected in this study were focused on specific exercises that are frequently used in knee rehabilitation. Future studies could also investigate how exergaming can be used for activities of daily living such as walking.

### Conclusions

This randomized controlled trial study explored the effectiveness of CKC and OKC game-based interventions in promoting motor learning with augmented feedback in healthy adults. The results demonstrated that augmented feedback significantly enhanced knee joint motor learning performance in both game contexts, highlighting its value as an intuitive and engaging form of feedback. Additionally, consistent muscle synergy patterns across tasks and groups suggest that the neuromuscular strategies used were stable and repeatable, reinforcing the reliability of augmented feedback game-based training for knee joint motor control. In the future, targeted populations such as clinical groups could be considered to test the effectiveness of rehabilitation.

## Appendix

Multimedia Appendix 1 CONSORT-eHEALTH checklist (V 1.6.1).

Multimedia Appendix 2 Complete overview of the results of the muscle synergy analysis.

## Abbreviations

AE: average error
AF: auditory feedback
AKA: actual knee angle
AME: average mean error
BF: biceps femoris
CG: control group
CKC: closed kinetic chain
CONSORT: Consolidated Standards of Reporting Trials
EG: experimental group
EMG: electromyography
IE: improvement of error trajectory
IMI: Intrinsic Motivation Inventory
IMU: Inertial Measurement Unit
KOR: knowledge of result
OKC: open kinetic chain
SENIAM: Surface Electromyography for the Non-Invasive Assessment of Muscle
TKA: target knee angle
UEQ: User Experience Questionnaire
UI: user interface
VAF: variance accounted for
VF: visual feedback
