# Supplementary material for: Enhancing Knee Joint Proprioception in Healthy Adults Through Exergame Training With Augmented Feedback: Randomized Controlled Pilot Trial
Source: JMIR Rehabil Assist Technol. 2026 Feb 26;13:e78525. doi: 10.2196/78525 (PMC12982958; doi:10.2196/78525)
Supplement: Multimedia Appendix 2 [file rehab_v13i1e78525_app2.docx]

For six synergies the NMF presents 6 different weights describing how the individual muscles contributed to the movement. The weight 1, 2, 3, 4, 5, and 6 are depicted in Figures S1-S6, respectively.


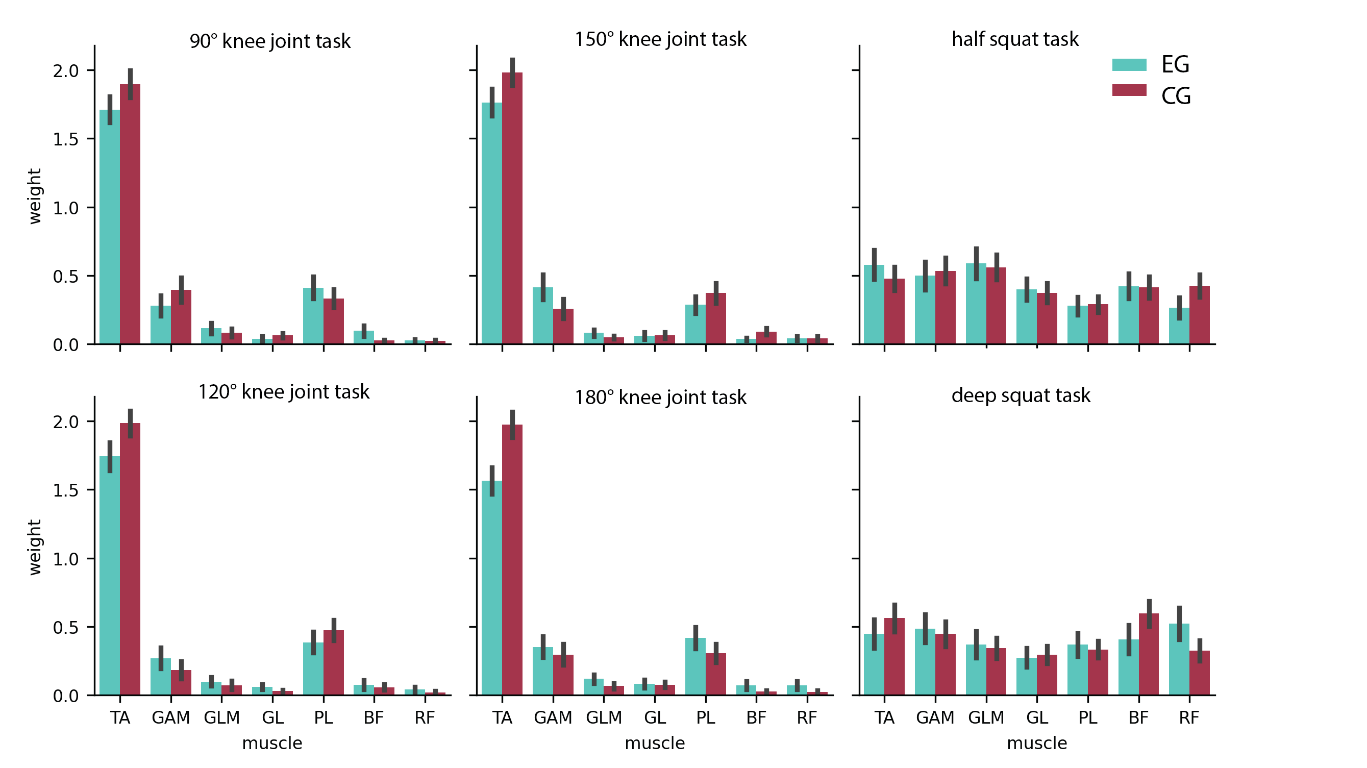


**Figure S1.** Synergy 1 weight analysis result.


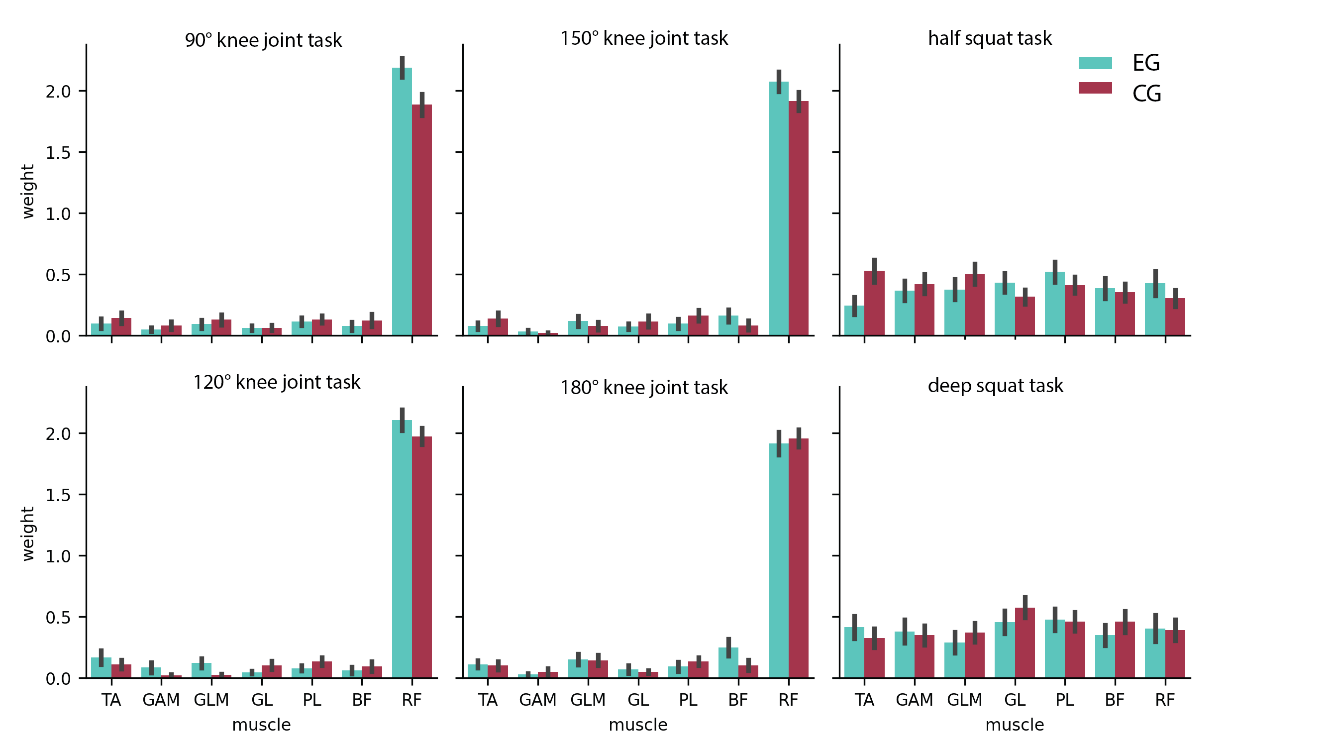


**Figure S2.** Synergy 2 weight analysis result.


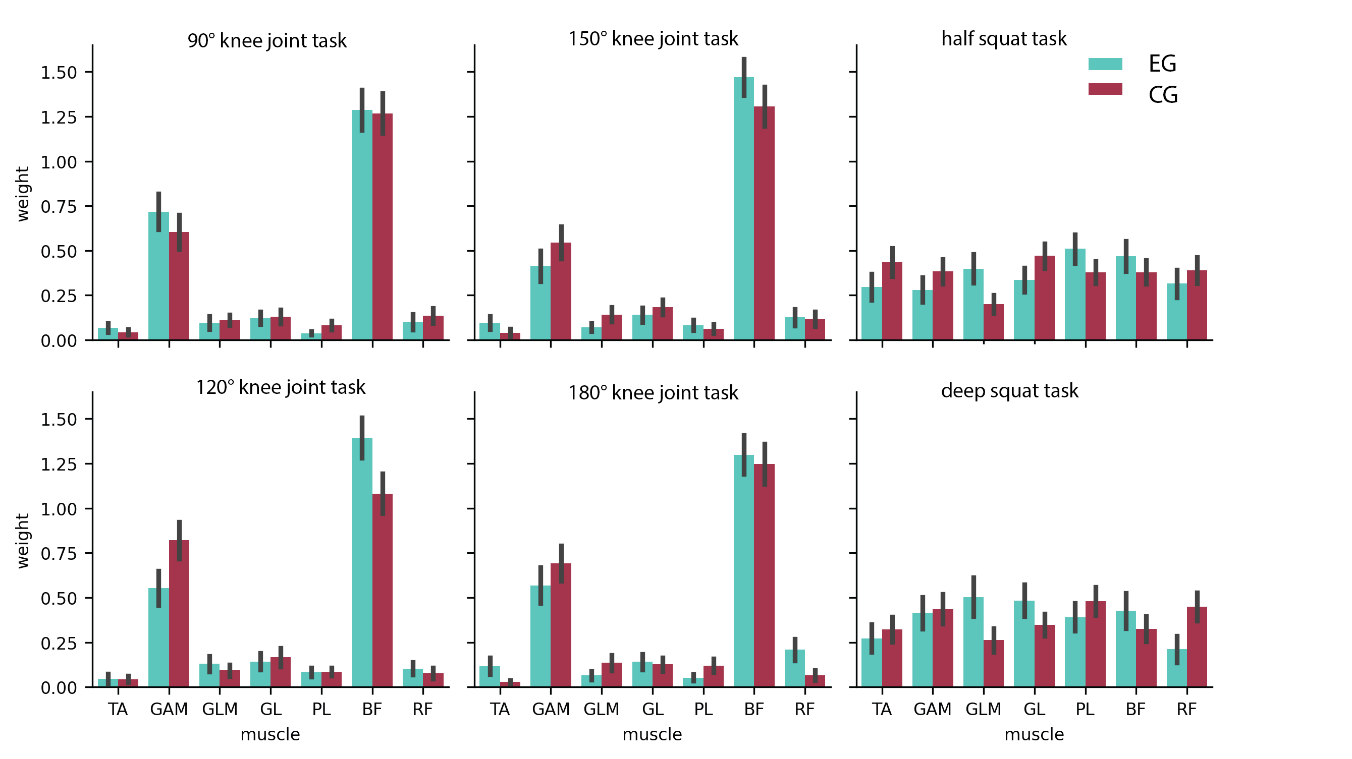


**Figure S3.** Synergy 3 weight analysis result.


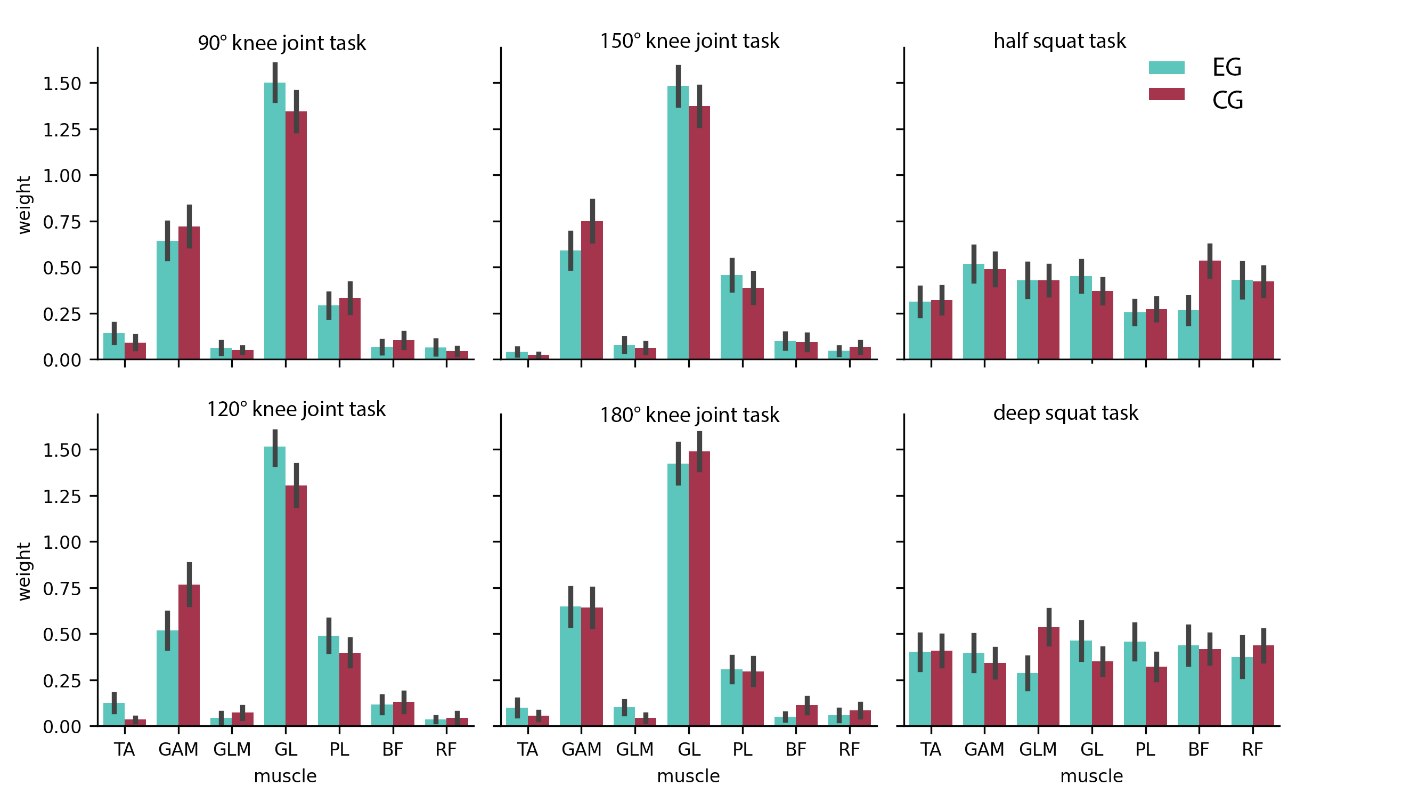


**Figure S4.** Synergy 4 weight analysis result.


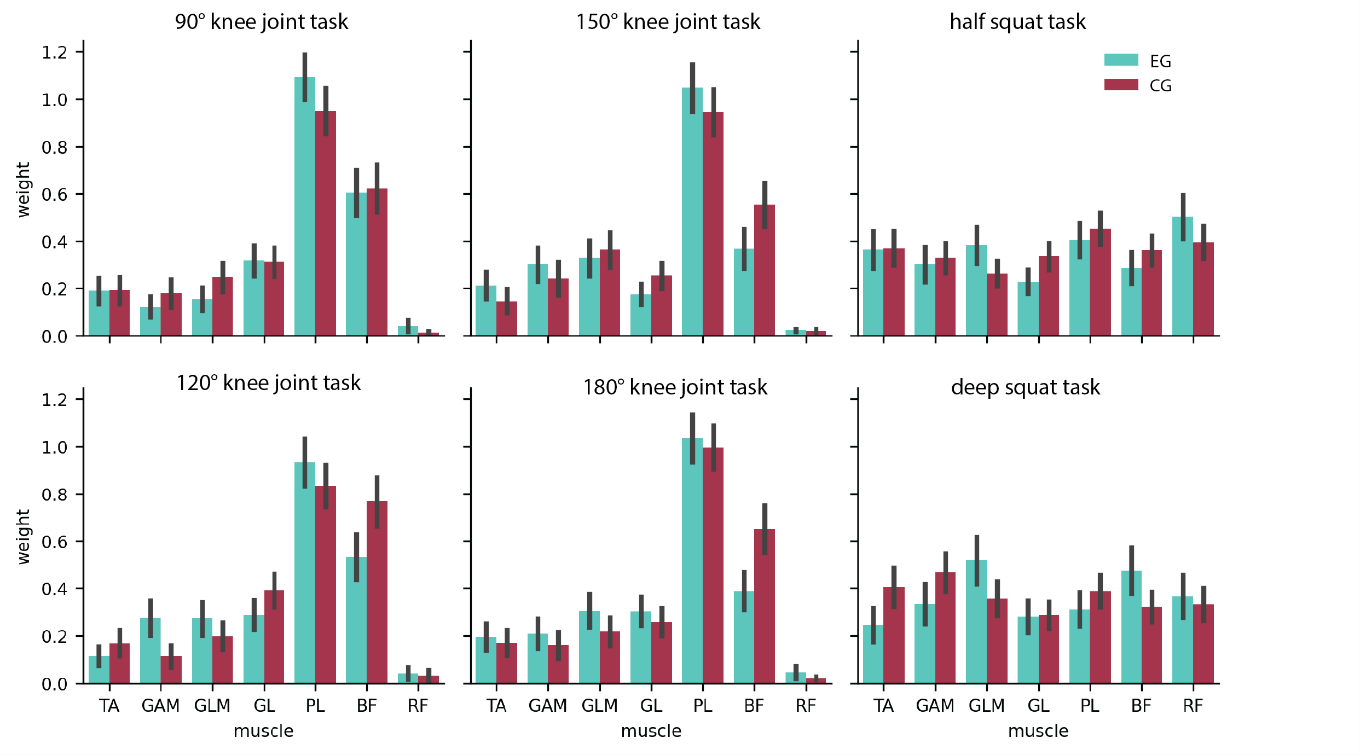


**Figure S5.** Synergy 5 weight analysis result.


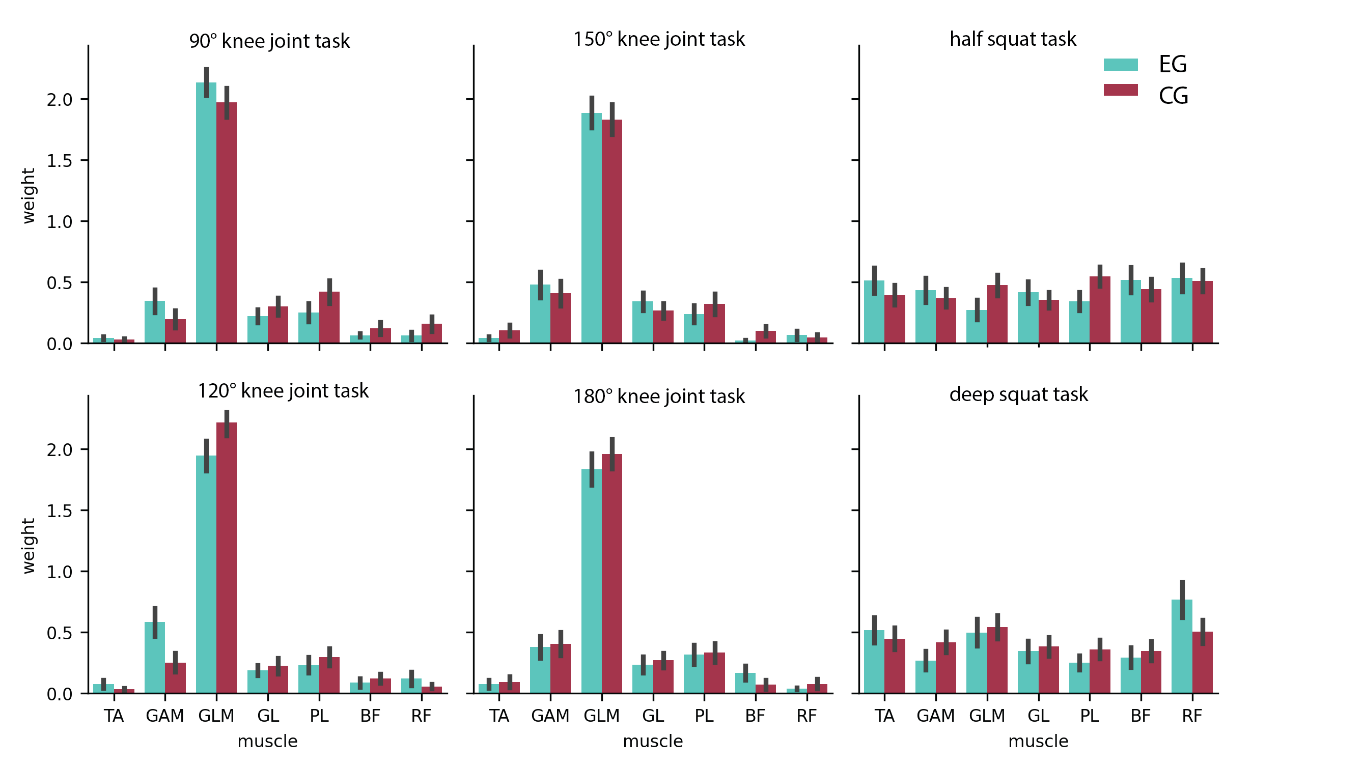


**Figure S6**. Synergy 6 weight analysis result.
